# Development and validation of an ultra-performance liquid chromatography quadrupole time of flight mass spectrometry method for rapid quantification of free amino acids in human urine

**DOI:** 10.1007/s00726-015-2076-0

**Published:** 2015-08-29

**Authors:** Richard Joyce, Viktorija Kuziene, Xin Zou, Xueting Wang, Frank Pullen, Ruey Leng Loo

**Affiliations:** Medway Metabonomics Research Group, Medway School of Pharmacy, Universities of Kent and Greenwich, Kent, UK; RJMS Consultancy, Rochester, Kent UK; Medway Metabonomics Research Group, School of Science, University of Greenwich, Kent, UK

**Keywords:** Free amino acids, Human urine, Absolute quantification, HILIC-UPLC-qTOF-MS

## Abstract

An ultra-performance liquid chromatography quadrupole time of flight mass spectrometry (UPLC-qTOF-MS) method using hydrophilic interaction liquid chromatography was developed and validated for simultaneous quantification of 18 free amino acids in urine with a total acquisition time including the column re-equilibration of less than 18 min per sample. This method involves simple sample preparation steps which consisted of 15 times dilution with acetonitrile to give a final composition of 25 % aqueous and 75 % acetonitrile without the need of any derivatization. The dynamic range for our calibration curve is approximately two orders of magnitude (120-fold from the lowest calibration curve point) with good linearity (*r*^2^ ≥ 0.995 for all amino acids). Good separation of all amino acids as well as good intra- and inter-day accuracy (<15 %) and precision (<15 %) were observed using three quality control samples at a concentration of low, medium and high range of the calibration curve. The limits of detection (LOD) and lower limit of quantification of our method were ranging from approximately 1–300 nM and 0.01–0.5 µM, respectively. The stability of amino acids in the prepared urine samples was found to be stable for 72 h at 4 °C, after one freeze thaw cycle and for up to 4 weeks at −80 °C. We have applied this method to quantify the content of 18 free amino acids in 646 urine samples from a dietary intervention study. We were able to quantify all 18 free amino acids in these urine samples, if they were present at a level above the LOD. We found our method to be reproducible (accuracy and precision were typically <10 % for QCL, QCM and QCH) and the relatively high sample throughput nature of this method potentially makes it a suitable alternative for the analysis of urine samples in clinical setting.

## Introduction

An optimal level of amino acids in the body is important for normal homeostasis. They are involved in the regulation of gene expression, cell metabolism and signalling and the biosynthesis of hormones (Wu 2009). The detection and quantification of free amino acids has been routinely applied for the diagnosis of new born with inborn error diseases (Piraud et al. 2011; Giordano et al. 2012). More recently, increasing literature has implicated the role of free amino acids in a number of diseases such as cardiovascular diseases (Batch et al. 2014), insulin resistance and type 2 diabetes (Lu et al. 2013), obesity (Morris et al. 2012; Wiklund et al. 2014), renal diseases (Batch et al. 2014; Kim et al. 2014; Niewczas et al. 2014), hepatic disorders (Fitian et al. 2014; Zhou et al. 2014) and a number of cancer-related disorders (Ma et al. 2014; Zang et al. 2014). The increased interest in free amino acids has prompted the need for a reliable and high-throughput simultaneous quantification of free amino acids in biological fluids.

Quantitative analysis of amino acids by traditional reverse-phase high-performance liquid chromatography (HPLC) is hampered by both the lack of a significant chromophore in many of their structures, which negates the use of UV detection, and by their high polarity, which leads to poor retention on reverse-phase columns. Quantification of physiological free amino acids in biofluids is often performed by ion-exchange chromatography with post-column derivatization using ninhydrin as chromophore (Moore et al. 1958; Spackman and Moore 1958; Waterval et al. 2009). However, this method typically involves time-consuming derivatization processes and often requires long chromatographic run times of about 2–3 h per sample, and therefore is not suitable for high throughput (Kaspar et al. 2009b). The method also suffers from lack of analyte specificity due to interference by co-eluting compounds and thus limits accurate quantitation for some amino acids such as methionine and phenylalanine (Dietzen et al. 2008). Moreover, this technique needs to be performed using dedicated equipment to ensure excellence in reproducibility (Waterval et al. 2009).

A variety of methods have been developed for profiling of amino acids in the body fluids. This includes methods such as capillary electrophoresis (Hirayama and Soga 2012; Lorenzo et al. 2013; Poinsot et al. 2014), gas chromatography–mass spectrometry (GC–MS) (Namera et al. 2002; Kaspar et al. 2009a; Kvitvang et al. 2011), liquid chromatography–mass spectrometry (LC–MS) (Chen et al. 2014; Le et al. 2014) and more sensitive and specific LC–MS/MS (Waterval et al. 2009; Giordano et al. 2012). In addition, several commercially available derivatization reagents (Fernández-Fígares et al. 2004; Armenta et al. 2009; Held et al. 2011; Salazar et al. 2012) and the use of volatile ion-pairing agents (Qu et al. 2002; Armstrong et al. 2007; Piraud et al. 2011) have also been applied to quantify the content of amino acids in biological samples with some success. Nonetheless, these methods are potentially limited by the instability in the derivatization process (Gu et al. 2007). Hydrophilic interaction liquid chromatography (HILIC) improves the retention of polar compounds and offers the potential for a successful method for the analysis of amino acids in complex biological matrices without the need of derivatization or ion-pairing agents. Indeed, the use of HILIC coupled with MS detection for the analysis of amino acids, in plant and cellular extracts, as well as in biological fluids such as serum, plasma and urine has been successfully applied (Langrock et al. 2006; Paglia et al. 2012; Yuan et al. 2012; Buiarelli et al. 2013; Guo et al. 2013; Zhou et al. 2013).

The guidance on bioanalytical method validation provided by the Food and Drug Administration (FDA) requires the demonstration of the reproducibility of a method in terms of its accuracy, precision and stability, when any modification to an existing method is made (US Food and Drug Administration 2001). In this study, we apply the same HILIC-based chromatographic method previously developed for the analysis of free amino acids in plant extracts as described by Guo et al. Instead of using a triple quadrupole LC–MS/MS method with targeted detection by multiple reaction monitoring (MRM) for quantitative analysis of these free amino acids, we apply a full scan LC–MS method using a qTOF. Here, we present and describe the development and validation of a UPLC-qTOF-MS method using HILIC conditions for simultaneous absolute quantification of free amino acids in human urine samples. We also applied this method to quantify the urine samples from a dietary intervention study.

## Materials and methods

### Chemicals and reagents

l-Phenylalanine (Phe), l-tryptophan (Trp), l-leucine (Leu), l-isoleucine (Ile), l-methionine (Met), l-valine (Val), l-alanine (Ala), l-threonine (Thr), l-glycine (Gly), l-serine (Ser), l-asparagine (Asn), l-aspartic acid (Asp), l-cysteine (Cys), l-arginine (Arg), l-histidine (Hit), l-lysine (Lys) were purchased from Sigma-Aldrich (Gillingham, Dorset, UK). l-Proline (Pro), l-glutamic acid (Glu), l-glutamine (Gln) and l-tyrosine (Tyr) were purchased from Fisher Scientific (Loughborough, UK). Their corresponding labelled amino acids, with the atom % deuteration shown in brackets, were purchased from Sigma-Aldrich for l-Phenyl-d5-alanine (Phe-d5, 98 %), l-tryptophan-indo-d_5_ (Trp-d5, 97 %), l-methionine-methyl-d_3_ (Met-d3, 98 %), l-valine-d_8_ (Val-d8, 98 %), l-proline-2,5,5-d_3_ (Pro-d3, 97 %), l-alanine-3,3,3-d_3_ (Ala-d3, 99 %), l-glycine-d_5_ (Gly-d5, 98 %), l-leucine-5,5,5-d_3_ (Leu-d3, 99 %), l-glutamic acid-2,3,3,4,4-d_5_ (Glu-d5, 98 %), l-asparagine-d_8_ (Asn-d8, 97 %), l-cysteine-2,3,3-d_3_ (Cys-d3, 98 %), l-histidine-(alpha-d, imidaxole,2,5-d_2_) hydrochloric monohydrated (Hit-d3, 97 %), l-tyrosine-phenyl-d_4_ (Tyr-d4, 98 %) and l-arginine-2,3,3,4,4,5,5-d_7_ hydrochloride (Arg-d7, 98 %); and CND Isotopes (QMX, Thaxted, UK) for l-isoleucine-2-d_1_ (Ile-d1, 98.9 %), l-threonine-2,3-d_2_ (Thr-d2, 98.8 %), l-serine-2,3,3-d_3_ (Ser-d3, 99.1 %) and l-glutamine-2,3,3,4,4-d_5_ (Gln-d5, 98.8 %). All reference amino acids had a purity >99 %. The HPLC grade acetonitrile, formic acid, and water were purchased from Sigma-Aldrich. Ammonium formate was purchased from Fisher Scientific. All chemicals and reagents were of appropriate analytical grades.

### Instrumentation and experimental conditions

The analyses of urine samples were performed on a Waters Synapt G2 UPLC-qTOF-MS (Waters Corporation, Wilmslow, UK) system consisting of a Waters binary solvent delivery system and an autosampler operating Masslynx acquisition software (Synapt, version 4.1 Waters, USA). The electrospray ionisation (ESI) used a capillary voltage of 0.8 kV for positive mode; cone voltage 15 V; source temperature 150 °C; desolvation temperature 350 °C; cone gas flow 50 L/h and desolvation gas flow 800 L/h. The scan range was m/z 50–600 Da and the mass spectrometer resolution was 10,000, enabling mass accuracy within 2 mDa. The Leu-enkephalin (m/z 556.2771) was used as the lock mass solution for accurate mass calibration during long analytical sequences to counteract the potential effect of calibration drift during the long analytical run time.

Five microliters of each sample was injected onto a Waters ACQUITY UPLC BEH amide column (1.7 µm, 2.1 mm × 100 mm) with a VanGuard HSS T3 (1.8 µM) pre-column. The HILIC chromatographic condition was based on Guo et al. (2013) with modifications to the gradient, aiming to improve the sensitivity and specificity of analytes with longer retention time. The mobile phases consisted of an aqueous phase (A), containing water, 10 mM ammonium formate, and 0.15 % formic acid and an organic phase (B), containing acetonitrile, 1 mM ammonium formate, and 0.15 % formic acid. The flow rate was fixed at 0.4 mL/min with a gradient elution that started at 15 % of A and increased linearly to 20 % in 6 min; 20–45 % A, 6–10 min; 45–55 % A, 10–12.5 min; and finally 55–95 % in 0.1 min. The LC flow was diverted to waste at 12.6 min and continued for 1.4 min of each run before the column was re-equilibrated in the initial condition for 2.9 min, in an attempt to flush highly polar urine components off the column and away from the mass spectrometer source. The column was maintained at 35 °C and the samples were maintained at a temperature of 4 °C prior to injection. A strong (20 % acetonitrile:80 % water) and weak (80 % acetonitrile:20 %water) needle wash was performed between each injection.

### Preparation of standard solutions and calibration standards

All stock solutions of labelled and non-labelled amino acids were prepared in water at 5 and 20 mM, respectively, except Tyr and Tyr-d4, both at 2 mM due to its low solubility. These non-labelled stock solutions were used to prepare calibration standards in diluent and pooled urine sample. Calibration ranges were determined based on the concentrations observed in the pooled QC urine sample. A final seven-point calibration standards at 0.1, 1.0, 2.0, 4.0, 6.0, 8.0, and 12.0 µM were prepared for Phe, Trp, Leu, Val and Thr. The calibration curves were at a factor of 0.1 for each of the above ranges for Arg and Glu; 0.2 for Met and Pro; 0.5 for Ile, Ser, Asn, and Lys; 1.5 for Gln; 2.5 for Tyr; 3 for Gly and Hit; and 5 for Ala. Cys and Asp were not added in the final calibration curve as neither was detected in the pooled QC sample. Moreover, Cys was found to be unstable and formed the dimer, cystine in the solution within 2 weeks and was hence not suitable for analysis of large-scale studies that involve long analytical runs whilst Asp gave a broad chromatographic peak.

A fixed amount of labelled amino acids were prepared at the same concentration of the third lowest point on the calibration curve for each amino acid, with a minimum concentration of 1 µM to ensure a good signal to noise ratio (S:N). All stock solutions were prepared and aliquoted and stored in glass vials at −20 °C until analyses.

### Preparation of human urine and quality control samples

All urine samples were deproteinized by the addition of acetonitrile (100 µL of urine: 1081.1 µL of acetonitrile) and centrifugation at 1800*g* for 10 min. The supernatant (708.7 µL) was added to 36 µL of labelled stock solution and 155.3 µL of water to give final solution of 900 µL with composition of 75 % acetonitrile: 25 % aqueous and a final dilution of 15 times for the urine samples and 25 times for labelled stock solutions. A QC sample was prepared using an equal part of all 646 urine samples from a dietary intervention study (see amino acid quantitation in human urine samples later for the description of this study). A volume of 6 mL of the QC sample was used to prepare each of the QC-low (QCL), QC-medium (QCM) and QC-high (QCH) samples, by spiking in a known concentration of each amino acid at 3, 30 and 75 times the lowest concentration on the calibration curve and they therefore represent a concentration in the low, medium and high range, respectively, of the calibration curve for each amino acid under investigation.

### Data analysis

The chromatographic data were processed using QuanLynx (v 4.1, Waters, USA). Automatic generation of extracted ion chromatograms (EICs) was achieved by an 8 mDa chromatogram mass window on the expected m/z and mass resolution of 10,000 for both the analytes and their deuterated analogues. For amino acids with incomplete baseline separation of chromatographic peaks, the integration was manually adjusted to ensure the non-labelled standard and labelled internal standard peaks were processed in a uniform manner to ensure consistent results.

The calibration curves were constructed by calculating the chromatographic peak area ratio of the analyte and internal standard (IS) for each amino acid and at each concentration level. The ratio was calculated using (IS concentration ÷ IS peak area) × analyte peak area. Linear regression analyses were performed using six replicates of the calibration curve data. The correlation coefficient (*r*) was calculated for each amino acid and considered as acceptable when the coefficient of determination *r*^2^ ≥ 0.99. The limit of detection (LOD) for each amino acid was determined using six replicates and was set at the chromatographic peak area compared to the blank sample with S:N > 3. The lower limit of quantification (LLOQ) was set at the lowest concentration of the calibration curve.

The matrix effect was determined by comparing the slope of the calibration curves, prepared in biological matrix and in diluents, and the matrix effect was considered negligible for slope ratios in the range 0.9–1.1 (Jia et al. 2011; Chen et al. 2012; Guo et al. 2013). The slope ratio = 1 implies no matrix effect of the mass spectrometer whilst ratio >1 indicates ionisation enhancement and <1 indicates suppression. The ratio of QCM and QCH was also measured against each calibration curve and similar acceptance criteria were applied. Carry-over was evaluated using both the highest point of the calibration curves and QCH, followed by diluents in three replicates.

Intra- and inter-day accuracy and precision were evaluated using replicates of each QCL, QCM and QCH on the same day and over a 4-week period. Accuracy was determined by calculating the percentage of deviation of the measured amount by the actual added amount, % accuracy = [mean measured amount − mean nominal amount ÷ mean nominal amount] × 100; and precision was calculated by the relative standard deviation (RSD) for each amino acid,  % precision = [standard deviation of mean measured amount ÷ mean measured amount] × 100. Precision and accuracy were considered acceptable when values were <20 % for QCL and <15 % for QCM and QCH for at least two-thirds of the replicates, based on the criteria outlined on the FDA guidelines (US Food and Drug Administration 2001). In addition, the stability of the amino acids in the urine samples was evaluated using a pooled QC urine sample under four different storage conditions: (1) freshly prepared; (2) short-term stability in 4 °C for 72 h; (3) 4 weeks storage at −80 °C; and (4) one freeze–thaw cycle after storage at −80 °C.

### Amino acid analysis by AccQ-Tag

Seventeen urine samples were analysed using HPLC system with a pre-column fluorescent derivatization reagent, AccQ-Tag, and were prepared following manufacturer standard protocols (Cohen and Michaud 1993). Data for all free amino acids were compared using Bland–Altman analysis where the mean difference between the two methods (UPLC-qTOF-MS and Accq-Tag) was obtained (Bland and Altman 1986). The mean deviation (%) of all measurements and mean difference in both absolute measured value (μM) and adjusted for urinary creatinine (μM/mM) as measured by Jaffe method were obtained. The relative total technical error of measurement (TEM) as expressed in % was calculated as $\frac{{\sqrt {\frac{{\mathop \sum \nolimits d^{2} }}{2N}} }}{{\bar{x}}}$ × 100 %, where *d* is the difference between measurements, *N* is the number of measurements made on each occasion and $\bar{x}$ is the mean of all samples values (Ulijaszek and Kerr 1999).

### Amino acids quantitation in human urine samples

Human urine samples were obtained from the OmniHeart Study. Details of the study design, aims and main outcomes of the study have been published (Appel et al. 2005; Carey et al. 2005; Furtado et al. 2008). Briefly, OmniHeart Study is a cross-over, three-period, randomised feeding trial where individuals were fed with three different healthy diets, each for 6 weeks. Urine samples were collected prior to the start of any dietary intervention and at the end of each 6-week intervention (*N* = 646 urine samples). Urine samples were transferred on dry ice to our central laboratory in Chatham, Kent for storage until analysis. All participants provided formal written consent and ethics approval was obtained from Johns Hopkins University medical institutions and Brigham Women’s Hospital.

We applied our method to quantify these 18 free amino acids using a random subset of human urine samples from the OmniHeart Study (*N* = 87) to evaluate the feasibility of this method on real biological matrix before applying the method on the remaining samples. This is important since our method had not previously been applied to human urine samples.

## Results and discussion

### Method development and optimization

This study aimed at the development and validation of a UPLC-qTOF-MS method using HILIC conditions for absolute quantitation of free amino acids in human urine samples. We assessed the optimal urine sample preparation process by adding fixed concentration of labelled compounds and diluting the pooled QC sample with water by a factor of 5, 10, 15, 20, 25, 50, 75 and 100 times but maintaining the final composition of 75 % acetonitrile:25 % aqueous. For most analytes, the results obtained were similar in the 15, 20 and 25 dilutions, with a maximum peak area for non-labelled compound in the 15-fold dilution, suggesting a degree of ion suppression at lower dilutions. Some compounds (Met, Val, Gly, Glu, Ser, Asn, Lys) were not detected in the QC pooled samples at dilutions greater than 15, suggesting that the absolute concentrations in these dilutions were approaching the LOD. All urine samples were, therefore, subsequently prepared using a 15-fold dilution.

**Fig. 1:**
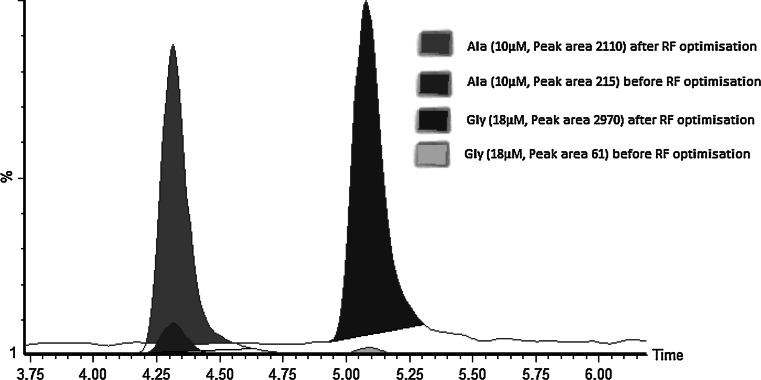
Effect of modification of the Quadrupole RF settings for Ala and Gly

We found the quadrupole RF settings were not optimal and this restricted the passage of low molecular mass ions (<100). We improved it by optimising the RF setting of the qTOF instrument, adjusting the RF offset settings to: source, 100 V; trap 140 V; IMS 50 V; and transfer 100 V. This resulted in increases in signal for Gly and Ala of approximately 50- and 10-fold, respectively (Fig. 1). The signal for other amino acids was not affected.

**Fig. 2:**
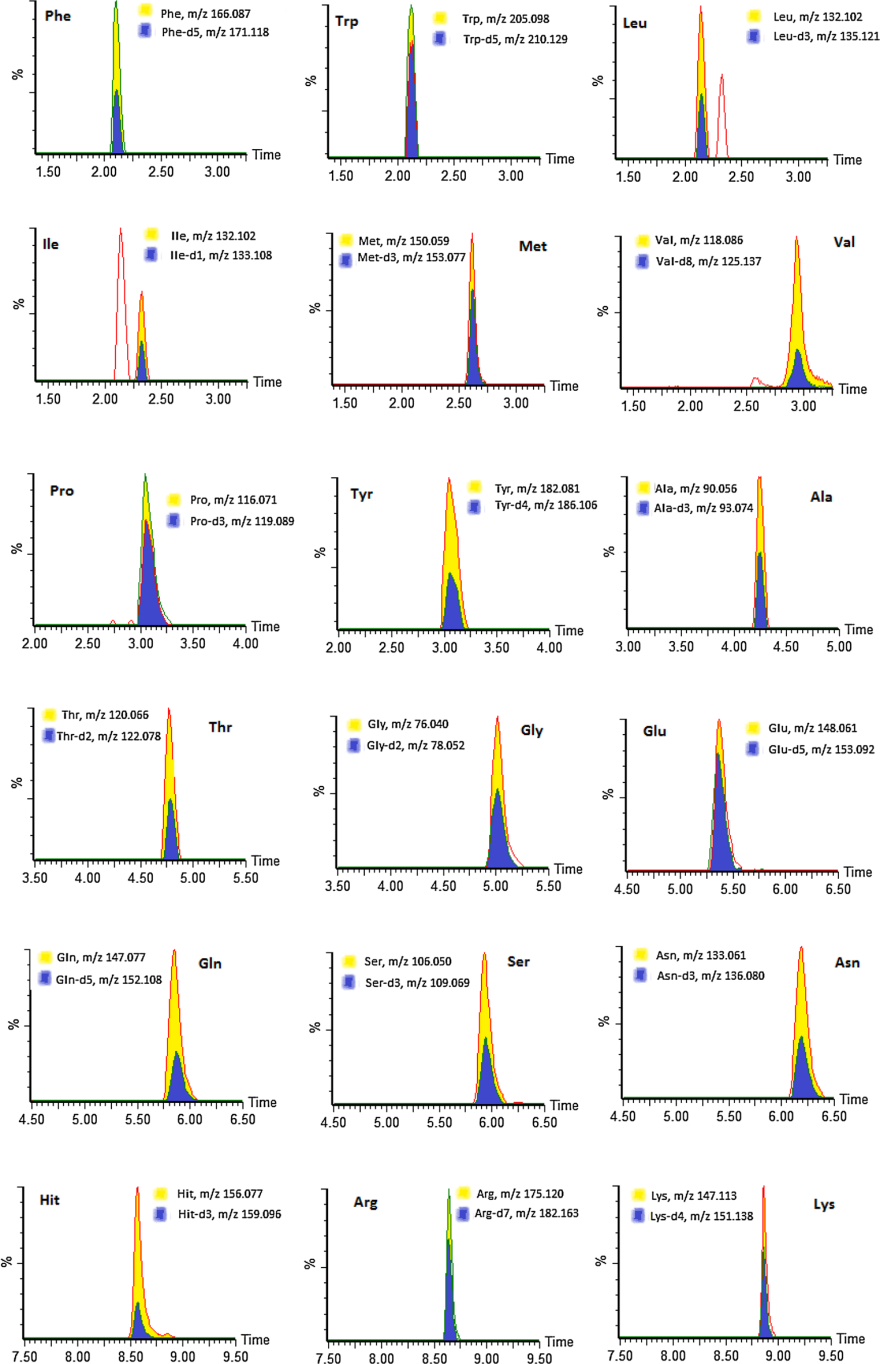
Typical UPLC accurate mass chromatograms of the ions extracted for non-labelled and deuterated amino acids in a standard mix.* Note* For l-glycine-d_5_, we monitored Gly-d_2_ and for l-asparagine-d_8_, we monitored Asn-d_3_ as the remainders of the deuterium are exchangeable

**Fig. 3:**
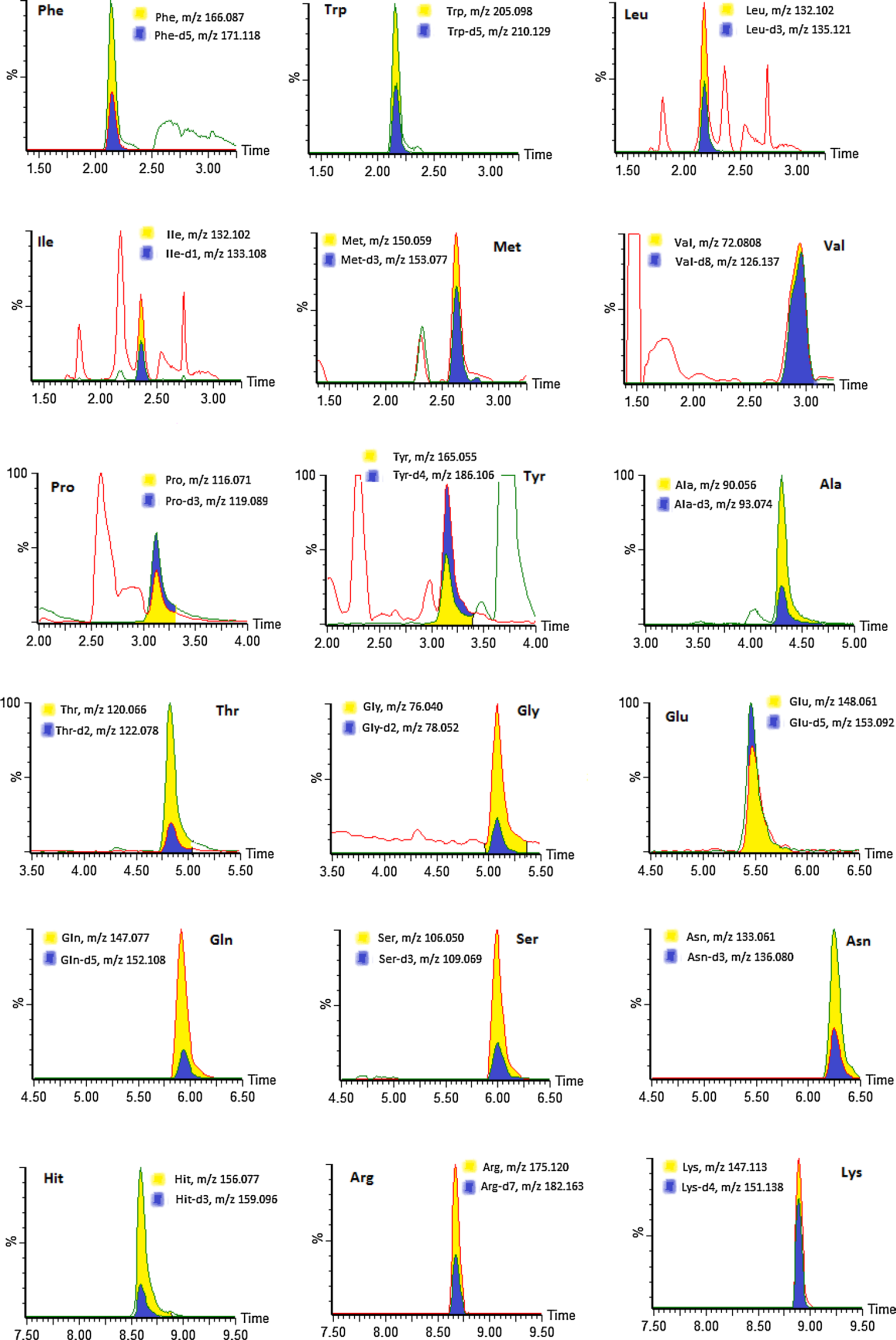
Typical UPLC accurate mass chromatograms of the ions extracted for non-labelled and deuterated amino acids in a urine sample

**Fig. 4:**
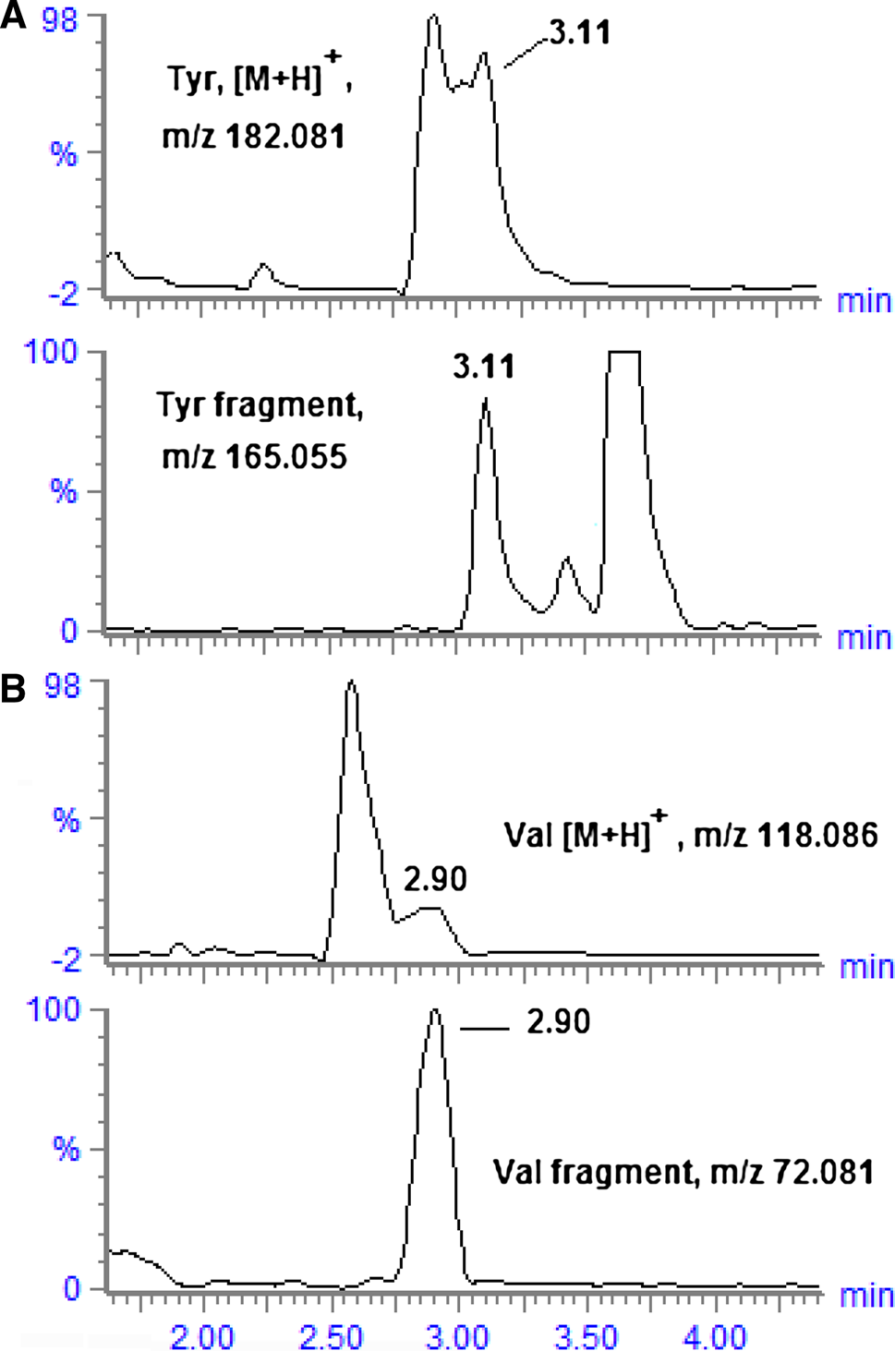
Extracted ion chromatograms that show interference from other closely eluting compounds in the urine sample for molecular ions of Tyr (panel **a**) and Val (panel **b**), and their corresponding fragment ion that are free from interference

Typical accurate mass chromatograms from a standard mix solution are shown in Fig. 2. All amino acids show satisfactory peak shape and minimal peak overlapping. The accurate mass chromatogram of urine samples is clearly more complex than the standard mix solution (Fig. 3). For all amino acids except Val and Tyr, we used the accurate mass for protonated molecular ion, [M + H]^+^ and the retention time to correctly identify and quantify the analytes. For Val and Tyr, the [M + H]^+^ ions show closely co-eluting compounds in the urine sample which prevent accurate quantification of Val and Tyr using [M + H]^+^. However, we were able to use fragment ions at m/z 72.081 ([M + H-HCO_2_H]^+^) and 165.056 ([M + H-NH_3_]^+^), respectively, to quantify the concentration of these two amino acids (Fig. 4). In both cases, the fragment ions are considerably less intense than the [M + H]^+^ ions, at approximately 4 and 15 times for Val and Tyr, respectively. This reduces the potential sensitivity and specificity of the method for these two compounds. Nevertheless, the combination of the detection of the correct fragment ion at the correct retention time provided adequate confidence on the use of the fragment ions. We recognise that the use of fragment ions is less satisfactory than the use of [M + H]^+^ ions but this was not possible due to interference from the urine samples.

### Selectivity, linearity, matrix effects and reproducibility of UPLC-qTOF-MS approach

Table 1 shows the results of the matrix effects, linearity, LOD, LLOQ and upper LOQ (ULOQ) of the method. The slope ratios of the calibration curves, QCM and QCH for all 18 analytes were between 0.9 and 1.1, indicating minimum matrix effects based on a 15-fold dilution. Since both types of calibration curves generated similar results, we have subsequently decided to prepare all calibration curves using diluent. The effects of carry-over were evaluated using the highest point of the calibration curve and QCH. No peak corresponding to labelled analytes was detected in the blank diluent (data not shown). The linearity, *r*^2^, was ≥0.995 for all amino acids and the dynamic range of the method, based on the calibration curve, was typically at two orders of magnitude (120-fold of the LLOQ). The intra- and inter-day accuracy and precision based on the QCL, QCM and QCH are shown in Table 2. The intra-day accuracy and precision were determined using four replicates of QCL, QCM and QCH in a single run. The inter-day analysis was performed using ten replicates over 4 weeks. We found our method fully complied with the FDA guidelines and with accuracy and precision of typically <15 % for QCL, QCM and QCH. The stability of the amino acids in urine samples was evaluated under four different conditions (Table 3). The urine samples remained stable for up to 72 h at fridge temperature (4 °C); for 4 weeks when stored at −80 °C; and when subjected to one freeze thaw cycle. These results were comparable to those from freshly prepared urine samples. The precision of all 19 replicates based on pooled QC samples for all four different conditions was generally <15 % for all amino acids. These results together with those from Table 2 thus show good reproducibility and providing confidence in our method.

**Table 1:** The matrix effects, coefficient of determination, limits of detection (LODs), lower limits of quantification (LLOQ) and upper limits of quantification (ULOQ) of each amino acid

Analyte	Matrix effect			Coefficient of determination (*r* ^2^)	LOD (nM)	LLOQ (µM)	ULOQ (µM)
	Slope ratio	Ratio QCM	Ratio QCH				
Phe	0.98	0.95	0.97	1.000	12.1	0.10	12.0
Trp	0.99	0.96	0.99	1.000	4.4	0.10	12.0
Leu	0.91	0.91	0.95	0.999	2.7	0.10	12.0
Ile	0.94	0.91	0.92	0.995	1.3	0.05	6.0
Met	0.97	0.94	0.95	0.997	0.7	0.02	2.4
Val	0.97	0.96	0.97	0.999	44.1	0.10	12.0
Pro	0.92	0.90	0.91	0.996	18.8	0.02	2.4
Tyr	1.05	0.95	1.05	0.997	21.3	0.25	30.0
Ala*	0.93	0.93	0.94	1.000	294.1	0.50	60.0
Thr	0.99	1.10	1.08	0.999	12.3	0.10	12.0
Gly*	0.95	0.93	0.91	0.999	145.2	0.30	36.0
Glu	0.95	0.91	0.91	0.995	6.4	0.01	1.2
Gln	1.00	1.03	1.04	1.000	23.9	0.15	18.0
Ser	0.99	0.97	0.98	0.996	23.4	0.05	6.0
Asn	1.03	0.98	0.99	0.998	20.8	0.05	6.0
Hit	0.98	1.02	0.99	0.999	10.4	0.30	36.0
Arg	1.03	1.00	1.10	0.999	5.9	0.01	1.2
Lys	1.03	1.01	1.02	0.996	8.3	0.05	6.0

LOD for Asp and Cys are 250 and 400 nM (fresh solution), respectively. No other data were available as neither was detected in the biological matrix

*LLOQ* lower limit of quantification. This corresponds to the lowest point in the calibration curve

*ULOQ* upper limit of quantification. This corresponds to the highest point in the calibration curve

* Results based on four replicates after re-optimising the RF setting for low mass molecules

**Table 2:** Intra- and inter-day accuracy and precision

Analyte	Intra-day reproducibility (over a single day)						Inter-day reproducibility (over 4 weeks)					
	QCL (*n* = 4)		QCM (*n* = 4)		QCH (*n* = 4)		QCL (*n* = 10)		QCM (*n* = 10)		QCH (*n* = 10)	
	Accuracy (bias, %)	Precision (RSD, %)	Accuracy (bias, %)	Precision (RSD, %)	Accuracy (bias, %)	Precision (RSD, %)	Accuracy (bias, %)	Precision (RSD, %)	Accuracy (bias, %)	Precision (RSD, %)	Accuracy (bias, %)	Precision (RSD, %)
Phe	10.0	11.6	1.5	2.0	−0.7	1.2	4.7	8.1	1.9	2.1	1.2	−0.8
Trp	9.5	9.3	3.9	1.5	2.4	3.6	6.4	7.6	3.6	2.3	3.0	2.7
Leu	1.7	2.6	−0.3	0.4	−3.5	1.9	0.9	2.5	2.2	4.8	−1.2	4.2
Ile	11.0	6.5	8.7	3.5	7.8	4.3	7.2	7.1	8.4	6.5	3.4	8.1
Met	17.4	12.4	1.2	6.4	2.5	6.6	12.1	7.6	1.2	5.0	−3.8	5.0
Val	5.6	3.6	10.2	3.0	5.5	4.2	7.9	3.2	8.2	3.0	4.6	3.3
Pro	7.5	9.1	6.9	5.3	7.7	1.3	7.0	8.1	0.4	8.8	1.9	6.6
Tyr	4.0	6.5	6.9	7.2	2.1	2.6	5.7	10.3	2.8	7.3	−4.7	7.9
Ala	0.3	2.6	3.2	1.0	0.2	2.2	−1.8	2.6	1.9	2.2	0.6	1.8
Thr	−7.7	12.8	−3.2	4.4	−1.8	4.5	0.5	10.5	1.9	5.1	−2.0	6.0
Gly	3.2	6.6	1.7	7.0	−3.2	10.4	2.4	5.5	4.1	6.5	−0.9	6.6
Glu	11.3	5.3	0.0	9.7	1.1	14.0	13.0	8.8	5.4	9.0	−0.3	9.3
Gln	7.8	2.3	6.6	9.3	−0.6	4.0	5.0	2.7	4.5	5.7	0.5	3.4
Ser	12.4	6.1	6.4	8.1	6.8	11.7	11.0	9.8	5.4	6.8	1.9	8.0
Asn	5.0	3.2	5.3	8.1	7.8	4.2	5.7	9.8	8.8	10.7	6.7	5.7
Hit	8.6	4.4	3.4	2.5	0.8	2.7	6.4	3.7	2.7	2.9	0.8	2.2
Arg	4.5	5.8	4.5	4.9	2.1	3.8	7.2	6.9	3.6	4.2	−0.7	4.6
Lys	7.0	6.2	5.3	2.6	1.5	1.5	4.8	7.6	2.0	3.9	0.1	2.0

**Table 3:** Stability of amino acids in the unspiked QC urine sample under different storage conditions

Analyte	Overall (*n* = 19)		Freshly prepared (*n* = 4)		Stored in 4 °C for up to 72 h (*n* = 9)		Stored in −80 °C for 4 weeks (*n* = 5)		One freeze thaw cycle (*n* = 5)	
	Mean measured concentration* (µM)	Precision (RSD, %)	Mean measured concentration* (µM)	Precision (RSD, %)	Mean measured concentration* (µM)	Precision (RSD, %)	Mean measured concentration* (µM)	Precision (RSD, %)	Mean measured concentration* (µM)	Precision (RSD, %)
Phe	2.17	2.3	2.13	0.9	2.17	2.5	2.18	3.1	2.17	1.2
Trp	3.70	2.1	3.76	1.1	3.74	1.8	3.63	1.6	3.70	2.1
Leu	1.53	5.2	1.50	1.4	1.55	4.1	1.43	2.1	1.58	3.6
Ile	0.65	8.2	0.66	9.2	0.67	5.9	0.58	2.8	0.68	4.7
Met	0.23	7.5	0.21	4.2	0.23	8.5	0.23	8.4	0.24	5.3
Val	1.55	5.8	1.53	4.9	1.58	5.3	1.49	2.5	1.55	7.7
Pro	0.29	5.9	0.31	4.1	0.30	5.3	0.28	6.5	0.28	4.0
Tyr	3.49	6.8	3.64	8.7	3.61	6.8	3.45	6.1	3.33	5.2
Ala	7.05	2.5	6.98	1.9	6.98	1.9	7.04	1.3	7.12	3.8
Thr	2.87	11.8	3.29	10.7	3.13	8.7	2.56	5.5	2.71	6.9
Gly	17.34	6.8	17.08	11.3	17.83	2.4	18.00	3.3	16.88	4.2
Glu	0.08	12.4	0.07	8.1	0.08	12.6	0.08	13.8	0.08	12.1
Gln	5.82	4.1	5.68	2.8	5.68	2.8	5.77	1.4	6.11	3.7
Ser	2.13	8.8	2.13	10.1	2.13	10.1	2.18	6.6	2.09	9.5
Asn	1.00	8.9	0.96	7.8	0.96	7.8	0.97	4.9	1.11	3.8
Hit	3.91	2.5	3.94	2.2	3.94	2.2	3.84	1.6	3.94	3.0
Arg	0.05	11.0	0.05	7.3	0.05	7.3	0.05	6.2	0.06	12.4
Lys	0.17	13.4	0.15	8.0	0.15	8.0	0.17	6.8	0.20	6.3

* The actual urinary concentration for each amino acid is 15 times of the reported measured concentration due to the 15-fold dilution of the urine sample during sample preparation

We found our method generally offered similar or better sensitivity than that obtained by other triple quadrupole methods using MRM detection and HILIC chromatography. The use of MRM enables the selection of single precursor and product ion for each compound of interest and therefore provides high specificity and selectivity for the quantification of each analyte. Specificity in our UPLC-qTOF-MS method is provided by the generation of accurate mass chromatograms and generally gave considerably lower LODs, between 20 and 50 times, for Arg, Glu, Lys, Asn, Hit, Ser and Thr but lower sensitivity for Pro and Ala (Guo et al. 2013; Yao et al. 2013). Our method also provided lower detection limits than other reported methods using time of flight mass analysers (Paglia et al. 2012). However, some recent advances in MS have applied the orbitrap mass spectrometer, operating in full scan mode (Nemkov et al. 2015), resulting in considerably better sensitivity. Domingues et al. applied triple quadrupole MRM method showing better sensitivity than our method, with LLOQ in the values of tens of nanomoles (Domingues et al. 2015). Others make use of derivatization agents (Armenta et al. 2009; Salazar et al. 2012) and ion-pairing agents (Gu et al. 2007; Le et al. 2014) for targeted analysis of polar metabolites including amino acids. Although these methods show better sensitivity than our method, the need of derivatization could introduce potential errors. Moreover, based on our previous experience, our initial assessment using an ion-pairing agent, perfluorocarboxylic acid, together with a reverse-phase UPLC-MS method for quantification of free amino acids has generated numerous challenges. We found the accumulation of perfluorocarboxylic acid in the instrument contaminated both the positive and negative ion electrospray which affects the sensitivity of the MS for this analysis and for analyses performed by other users on the same instrument. The accumulation of perfluorooctanoic acid in the column also affected the properties of column and led to instability of retention time for several amino acids (data not shown). These limit the application of the method and are particularly not suitable for use in our institution where the instrument is used by other users for different types of analyses on a daily basis. As a consequence, we applied a HILIC method that does not require any derivatization or ion-pairing agents. Our method using the qTOF at high resolution enables accurate mass scanning across m/z 50–600. This enables targeted analysis, of all 18 free amino acids by spiking in deuterated internal standards, and untargeted analysis of other compounds present in the analytical samples in the same analytical run. We considered that the latter feature offers a significant advantage over some of the existing methods as these data may be further analysed to extract additional useful information. The data on untargeted analysis are beyond the scope of this paper and therefore will not be discussed further.

The throughput of our method is good, 18 min per sample including column re-equilibration time. Our throughput may not be as high compared to some of the methods discussed here, typically with a total acquisition of <10 min (Buiarelli et al. 2013; Nemkov et al. 2015), whilst Nemkov et al. by far is the quickest method with a 3-min acquisition time (Nemkov et al. 2015).

### Comparison of amino acid analysis with AccQ-Tag

The data obtained by the AccQ-Tag method were unsatisfactory for most amino acids. Throughout the chromatogram, large numbers of compounds in the urine were found to co-elute with the free amino acids. In addition, co-elution amongst free amino acids was also observed, such as Ser, Asn and Gln. Manual processing on all the data had enabled confident quantification of four amino acids, namely Phe, Trp, Leu and Ile. We subsequently calculated the TEM for these four amino acids and found the TEM was typically <26 % (Ile 15.7 %, Phe 17.2 %, Leu 17.7 % and Trp 25.6 %).

**Fig. 5:**
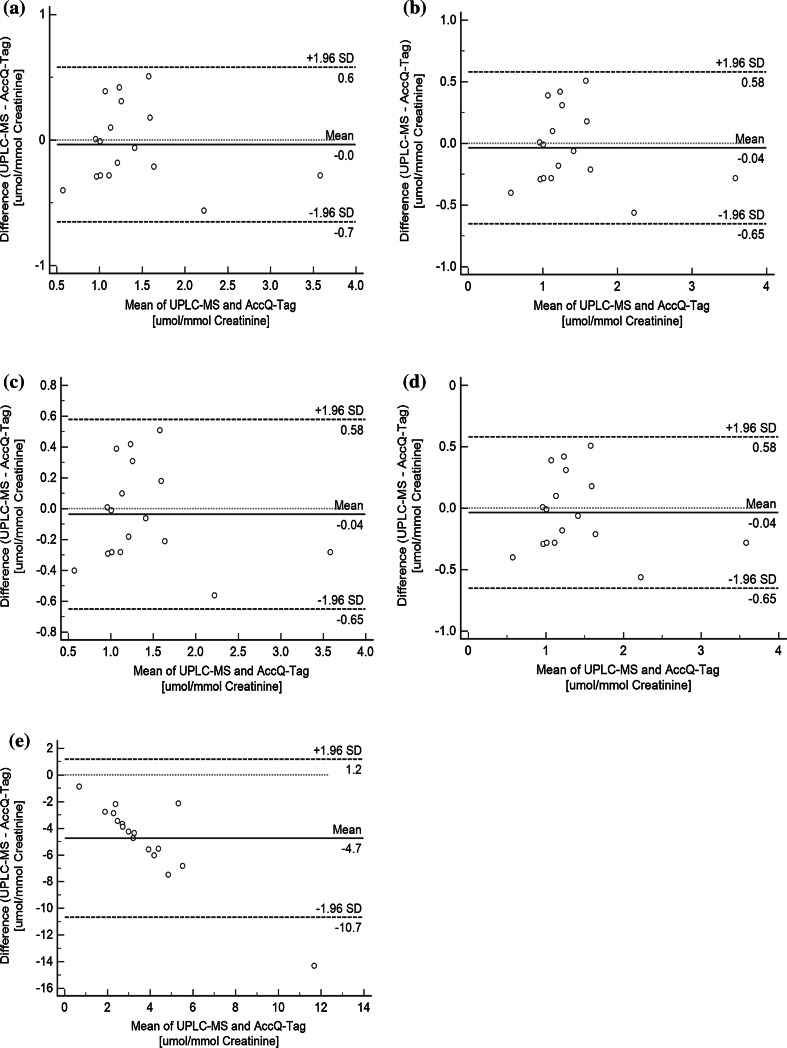
Bland–Altman plots for amino acids showing agreement between UPLC-MS and AccQ-Tag methods for **a** Phe, **b** Trp, **c** Ile, **d** Leu, and **e** Asn

Bland–Altman plots that assess the agreement between two different measurement techniques were plotted for Phe, Try, Leu and Ile using both the absolute measured value (μM) and adjusted for urinary creatinine (μM/mM). The mean difference between two measurements and limits of agreement within ±1.96 standard deviation (SD) of the differences are included as references. As the results were similar for both the mean absolute measured values and the adjusted for urinary creatinine values, we only presented the results based on the adjusted for urinary creatinine (Fig. 5). In general, we found all four amino acids showed good agreement between the two methods. The mean difference to the mean concentration for all measurements between the two methods was the lowest for Ile (2.9 %), followed by Phe (11.4 %), Trp (12.1 %) and the highest for Leu (16.1 %). Moreover, individual measurement differences of the four amino acids scatter randomly with no apparent systematic error being detected. We also plotted the Bland–Altman plots for the remaining 14 amino acids and found these amino acids showed lower concentration values for UPLC-qTOF-MS method. We found the mean differences were proportionately more negative with higher concentration of the analyte indicating some systematic errors. This is in compliance with our observations where co-elution was observed in AccQ-Tag method. An exemplar of this is shown using Asn, Fig. 5e.

As the majority of the free amino acids were not validated using the AccQ-Tag method, we then validated our method using a NIST certified amino acids standard containing 17 amino acids at 2.5 mM each. This NIST standard contains all the amino acid of our interest except Trp, Gln and Asn. However, as the Bland–Altman plots show good agreement between AccQ-Tag and UPLC-qTOF-MS for Trp, we deemed the results of our UPLC-qTOF-MS were acceptable for Trp. However, we were unable to further validate the results for Gln and Asn. The remaining 15 amino acids (Ala, Arg, Glu, Gly, Hit, Ile, Leu, Lys, Met, Phe, Pro, Ser, Thr, Tyr and Val) were analysed by UPLC-qTOF-MS method after serial dilution with water to give concentrations of 0.8 and 5 µM. These concentrations were chosen to allow the majority of amino acids to have at least two concentration points that fall within our standard calibration curve for each compound. In addition, we also extended the calibration curves to 14-point calibration curve for each amino acid to accommodate the compounds that were above or below the initial seven-point calibration curve. Each NIST standard (0.8 and 5 µM) was analysed in replicates of five and the results are presented in Table 4. The majority of the amino acids show good accuracy and precision (generally <5 %), again were well within the required FDA guidelines for developing an analytical method for the analysis of biofluids. Based on these results, we found the dynamic range of our calibration curve could potentially be extended considerably up to three orders magnitude (up to 2400-fold) for six of the amino acids. The dynamic range for Tyr, Pro, Thr, Glu, Ser, Lys and Arg was extended to 300-fold whilst for Gly and Hit, they were extended to 600-fold. The linearity for this extended dynamic range remains good with *r*^2^ ≥ 0.993 (Table 4). This extended dynamic range is similar to that reported for triple quadrupole methods of 2–3 orders of magnitude (Guo et al. 2013).

**Table 4:** Accuracy, precision and dynamic range for the quantification of amino acids in NIST certified standards (five replicates)

Analyte	NIST standard (0.8 µM)				NIST standard (5 µM)				Dynamic range^c^		
	Mean measured concentration (µM)	SD	Precision (RSD, %)	Accuracy (bias, %)	Mean measured concentration (µM)	SD	Precision (RSD, %)	Accuracy (bias, %)	LLOQ (µM)	ULOQ (µM)	Coefficient of determination (*r* ^2^)
Phe	0.76	0.02	2.1	−5.2	5.09	0.07	1.4	1.7	0.025	30.0	0.998
Leu	0.72	0.02	3.2	−9.6	5.04	0.13	2.6	0.7	0.0125	30.0	0.997
Ile	0.76	0.02	2.2	−4.6	4.77	0.21	4.4	−4.6	0.0125	30.0	0.995
Met^a^	0.77	0.02	2.2	−4.0	5.01	0.16	3.3	0.2	0.00625	15.0	0.999
Val	0.8	0.01	1.4	−0.6	5.12	0.03	0.6	2.4	0.0025	6.0	0.993
Pro^a^	0.77	0.01	0.8	−3.6	5.11	0.12	2.3	2.2	0.1	30.0	0.996
Tyr	0.77	0.03	3.9	−3.7	4.84	0.13	2.6	−3.2	0.02	6.0	0.998
Ala^b^	0.77	0.02	2.7	−3.5	4.61	0.06	1.2	−7.9	0.0625	75.0	0.996
Thr	0.78	0.01	1.3	−2.4	4.95	0.15	3.1	−1.0	0.5	150.0	0.997
Gly	0.8	0.03	3.2	−0.5	5.07	0.14	2.9	1.4	0.05	30.0	0.999
Glu^b^	0.76	0.02	2.3	−5.6	4.61	0.11	2.3	−7.8	0.3	90.0	0.997
Ser	0.81	0.03	3.1	1.4	4.95	0.10	2.1	−1.0	0.01	3.0	0.997
Hit	0.74	0.01	1.4	−7.0	4.54	0.11	2.3	−9.1	0.075	45.0	0.997
Arg^a^	0.8	0.02	2.0	−0.3	5.25	0.09	1.8	5.0	0.05	15.0	0.999
Lys	0.78	0.02	2.8	−1.9	4.91	0.10	2.1	−1.8	0.05	15.0	0.999

^a^The NIST Standard at 5 µM is above the range of the standard calibration curve for Met, Pro, Glu and Arg. These compounds were also measured against NIST Standard at 0.025 µM. The mean, standard deviation, precision and accuracy were Met (0.025 µM, 0.0016, 6.4 and 1.7 %), Pro (0.025 µM, 0.0014, 5.7 and 0.9 %), Glu (0.028 µM, 0.0011, 6.4 and 12.3 %) and Arg (0.027 µM, 0.0033, 12.1 and 9.0 %)

^b^A higher NIST Standard at 50 µM was also measured for Ala due to its higher calibration range. The mean, standard deviation, precision and accuracy for Ala were 52 µM, 0.4614, 0.9 and 4.0 %

^c^Standard calibration curves were created with a minimum concentration of 0.625 nM and a maximum concentration of 150 µM. The LLOQ was taken as the concentration of the standard compound that gave a S:N > 5 and the ULOQ was the highest concentration of the calibration curve

### Clinical application

Having validated our UPLC-qTOF-MS method using the NIST standard and to show utility of this method in real samples, we analysed 87 urine samples randomly selected from the OmniHeart study. We were able to detect and quantify all 18 free amino acids in all urine samples. The levels of concentration detected were generally within the ranges of the calibration curve used here in this paper. In our experimental design, we included the analysis of a QC pooled sample in every 12th sample and included three replicates of QCL, QCM and QCH as well as a seven-point calibration standards. Using this design, we were able to complete the analysis of 87 samples within 2 days. This is quicker than existing methods such as the AccQ-Tag and ninhydrin methods where these methods typically required 2–3 h analysis time per sample. We found the accuracy and precision of the QCL, QCM and QCH fulfilled current FDA requirements (Table 5). We then applied this method to quantify the remaining 559 samples. These were analysed over 4 batches, with some batches containing up to 192 samples. Each batch was analysed without cleaning the instrument. A total of 45 QCL, QCM and QCH samples were acquired. We use a 25 mDa chromatogram mass window to extract the EICs to mitigate any effects of mass calibration drift in the instrument over long (up to 90 h for some batches) chromatographic runs. These QC samples were used to evaluate the inter-batch precision and accuracy of our method. We found the inter-batch accuracy and precision was typically <10 % for QCL and QCM and <5 % for QCH (Table 6).

**Table 5:** Application of UPLC-qTOF-MS for the analysis of 87 human urine samples acquired in one batch

Analyte	QCL (*n* = 6)			QCM (*n* = 6)			QCH (*n* = 6)		
	Mean measured concentration* (µM)	Accuracy (bias, %)	Precision (RSD, %)	Mean measured concentration* (µM)	Accuracy (bias, %)	Precision (RSD, %)	Mean measured concentration* (µM)	Accuracy (bias, %)	Precision (RSD, %)
Phe	2.52	1.7	3.9	5.29	2.3	2.0	9.60	−0.8	1.3
Trp	4.11	3.8	4.3	6.87	3.1	2.4	11.53	3.3	2.2
Leu	1.82	0.5	2.6	4.68	3.8	5.6	9.04	0.3	4.7
Ile	0.81	3.8	2.9	2.30	7.8	8.1	4.40	0.3	9.0
Met	0.32	10.4	3.7	0.84	1.6	4.1	1.65	−4.5	4.2
Val	1.99	9.3	2.5	4.82	6.8	2.4	9.38	4.1	2.8
Pro	0.36	5.0	3.7	0.84	−4.7	4.2	1.74	−2.4	3.8
Tyr	4.38	5.6	12.7	10.85	−0.4	4.6	20.07	−9.4	5.4
Ala	8.33	−2.9	2.5	22.35	1.2	2.7	45.00	0.9	1.7
Thr	3.02	2.9	6.9	5.83	3.5	5.1	9.79	−3.3	4.8
Gly	18.60	1.4	4.4	27.88	5.5	6.7	40.15	0.5	3.2
Glu	0.13	16.1	7.9	0.41	9.4	6.1	0.82	−1.0	6.2
Gln	6.66	4.3	3.0	10.83	3.8	2.7	17.45	1.5	1.9
Ser	2.54	11.1	11.9	3.83	5.3	6.5	5.83	−0.9	3.3
Asn	1.29	7.9	9.6	2.85	12.0	10.6	5.10	6.4	6.8
Hit	5.02	4.7	2.1	13.18	2.3	3.2	26.62	0.9	2.1
Arg	0.09	9.6	5.8	0.36	3.1	4.1	0.78	−2.5	4.6
Lys	0.35	4.8	8.5	1.69	0.1	4.1	3.91	−0.8	2.1

* The actual urinary concentration for each amino acid is 15 times of the reported measured concentration due to the 15-fold dilution of the urine sample during sample preparation

**Table 6:** Application of UPLC-qTOF-MS for the analysis of 646 human urine samples, analysed over a total of 5 batches

Analyte	QCL (*n* = 45)			QCM (*n* = 45)			QCH (*n* = 45)		
	Mean measured concentration* (µM)	Accuracy (bias, %)	Precision (RSD, %)	Mean measured concentration* (µM)	Accuracy (bias, %)	Precision (RSD, %)	Mean measured concentration* (µM)	Accuracy (bias, %)	Precision (RSD, %)
Phe	2.56	3.7	3.3	5.32	3.0	2.0	9.69	0.2	1.7
Trp	4.25	3.0	4.0	7.05	3.2	2.9	11.57	2.1	2.5
Leu	1.84	0.8	3.6	4.66	3.0	3.5	9.17	1.5	3.1
Ile	0.82	2.3	5.5	2.32	7.5	5.0	4.44	0.8	4.4
Met	0.32	5.0	6.8	0.84	−0.3	4.4	1.68	−3.8	3.5
Val	2.05	7.4	7.2	4.83	4.7	4.0	9.42	3.4	2.4
Pro	0.38	3.1	6.1	0.90	−0.6	8.0	1.77	−2.0	5.1
Tyr	4.55	3.4	7.2	11.16	0.1	5.5	21.58	−3.6	4.5
Ala	8.54	−1.2	2.6	22.38	1.1	2.9	45.10	1.0	2.6
Thr	3.05	0.7	4.4	5.86	2.2	3.2	10.07	−1.6	3.1
Gly	18.81	2.8	2.7	27.62	4.6	3.7	39.82	−0.2	3.1
Glu	0.12	9.6	8.8	0.41	7.5	5.5	0.84	1.3	4.9
Gln	6.53	4.2	2.5	10.72	3.9	2.5	17.33	1.6	2.6
Ser	2.36	4.7	6.4	3.74	3.8	6.1	5.76	−1.5	4.2
Asn	1.21	4.0	6.5	2.63	4.4	7.1	4.90	2.9	4.8
Hit	4.98	5.0	2.3	13.19	2.7	2.5	26.42	0.3	2.4
Arg	0.09	8.9	5.3	0.36	1.6	3.8	0.79	−1.0	3.3
Lys	0.34	3.5	6.5	1.66	−0.9	4.1	3.85	−1.8	4.2

* The actual urinary concentration for each amino acid is 15 times of the reported measured concentration due to the 15-fold dilution of the urine sample during sample preparation

## Conclusion

A HILIC-based chromatographic method was adapted to a UPLC-qTOF platform for simultaneous quantification of 18 free amino acids in human urine samples. The UPLC-qTOF-MS method involves a facile sample preparation step which involves dilution with acetonitrile without the need of any derivatization. The method has been successfully applied for the analysis of human urine samples obtained from human volunteers with good accuracy and precision that passed all the FDA requirements. This demonstrates our approach a potential valuable tool to provide high-quality targeted analysis for the characterization of free amino acids in the urine samples. Moreover, the UPLC-qTOF-MS method also offers an added advantage whereby additional untargeted data collected during the analysis may be further analysed to extract useful information.
